# Peripheral Blood Smears in COVID-19: A Response

**DOI:** 10.4274/tjh.galenos.2020.2020.0723

**Published:** 2021-02-25

**Authors:** Maryame Ahnach

**Affiliations:** 1 Cheikh Khalifa International University Hospital, Mohammed VI University of Health Sciences, Department of Hematology, Casablanca, Morocco

**Keywords:** Blood cells, COVID-19, Infectious diseases

## To the Editor,

We thank Mungmunpuntipantip and Wiwanitkit for their comment on our publication on peripheral blood smears in coronavirus disease-19 (COVID-19) and we would like to reply.

In our experience of patients with COVID-19 admitted to the Cheikh Khalifa International University Hospital, we found 30.3% of severe cases with a 57.9% comorbidity rate and 23.3% rate of other diseases, but no cases of parasitic infection or allergic manifestations during hospital admission [1]. Consistent with the current evidence, asthma and allergic comorbidities do not seem to increase the risk for poor outcomes in cases of COVID-19 [2]. However, many publications report the diagnostic and prognostic value of eosinopenia in COVID-19 infection [3,4]. The pathophysiology for eosinopenia may be multifactorial, including inhibition of eosinophil egress from the bone marrow, blockade of eosinophilopoiesis, reduced expression of chemokine receptors/adhesion factors, and/or direct eosinophil apoptosis induced by type 1 interferons released during the acute infection [2]. The role of eosinophils remains unclear, but this does not exclude their impact on blood cell morphology. Regarding neutrophil lineages, Zini et al. [5] made a similar observation about dysmorphic granulocytes.

COVID-19 causes inflammatory and thrombotic complications, which can lead to both quantitative and qualitative hematologic abnormalities. Studies remain limited and we need a large number of cases to analyze the changes in blood cell morphology during COVID-19.
